# Multi-Element Analysis and Origin Discrimination of *Panax notoginseng* Based on Inductively Coupled Plasma Tandem Mass Spectrometry (ICP-MS/MS)

**DOI:** 10.3390/molecules27092982

**Published:** 2022-05-06

**Authors:** Chao Ji, Jinyu Liu, Qin Zhang, Juan Li, Zhiqiang Wu, Xingyu Wang, Yuxin Xie, Jiangchao Zhao, Rui Shi, Xing Ma, Mohammad Rizwan Khan, Rosa Busquets, Xiahong He, Youyong Zhu, Shusheng Zhu, Wenjie Zheng

**Affiliations:** 1 State Key Laboratory for Conservation and Utilization of Bio-Resources in Yunnan, National Engineering Research Center for Applied Technology of Agricultural Biodiversity, College of Plant Protection, Yunnan Agricultural University, Kunming 650201, China; jichao1100@163.com (C.J.); dianchahuyihao@163.com (J.L.); hexiahong@hotmail.com (X.H.); yyzhu@ynau.edu.cn (Y.Z.); 2 Laboratory for Quality Control and Traceability of Food and Agricultural Products, Tianjin Normal University, Tianjin 300387, China; 17854265918@163.com (Q.Z.); lijuandk0916@163.com (J.L.); wuzhiqiang314@126.com (Z.W.); wangxingyu100198@163.com (X.W.); wugeheshimo@163.com (Y.X.); 3 Department of Animal Science, Division of Agriculture, University of Arkansas, Fayetteville, AR 72701, USA; jzhao77@uark.edu; 4 Key Laboratory for Forest Resources Conservation and Utilization in the Southwest Mountains of China, Ministry of Education, Southwest Landscape Architecture Engineering Research Center of National Forestry and Grassland Administration, Southwest Forestry University, Kunming 650224, China; shirui@swfu.edu.cn; 5 The Animal, Plant & Foodstuff Inspection Center, Tianjin Customs, Tianjin 300387, China; xingma2005@126.com; 6 Department of Chemistry, College of Science, King Saud University, Riyadh 11451, Saudi Arabia; mrkhan@ksu.edu.sa; 7 School of Life Sciences, Pharmacy and Chemistry, Kingston University London, Penrhyn Road, Kingston upon Thames KT1 2EE, UK; r.busquets@kingston.ac.uk

**Keywords:** *Panax notoginseng*, ICP-MS/MS, multi-element, origin discrimination, cultivation model discrimination

## Abstract

*Panax notoginseng* is an important functional health product, and has been used worldwide because of a wide range of pharmacological activities, of which the taproot is the main edible or medicinal part. However, the technologies for origin discrimination still need to be further studied. In this study, an ICP-MS/MS method for the accurate determination of 49 elements was established, whereby the instrumental detection limits (LODs) were between 0.0003 and 7.716 mg/kg, whereas the quantification limits (LOQs) were between 0.0011 and 25.7202 mg/kg, recovery of the method was in the range of 85.82% to 104.98%, and the relative standard deviations (RSDs) were lower than 10%. Based on the content of multi-element in *P. notoginseng* (total of 89 mixed samples), the discriminant models of origins and cultivation models were accurately determined by the neural networks (prediction accuracy was 0.9259 and area under ROC curve was 0.9750) and the support vector machine algorithm (both 1.0000), respectively. The discriminant models established in this study could be used to support transparency and traceability of supply chains of *P. notoginseng* and thus avoid the fraud of geographic identification.

## 1. Introduction

Sanqi (*Panax notoginseng* (Burk.) F. H. Chen) is a perennial herb belonging to the genus *Panax* and is considered an evolutionary remnant that originated in areas ranging from East Asia to North America in the tertiary tropical mountainous area 25 million years ago. Today, its modern distribution center is located in Yunnan, China [1], and it has been widely used in medicine, health care, cosmetics, and other industries. *P. notoginseng* has a wide range of pharmacological activities, including cardiovascular protection [2], anti-atherosclerotic activity [3], and anti-tumor activity [4], with the cardiovascular impact being the most prominent. Therefore, it is a key component of many Chinese medicines which has been used worldwide and is of significant economic value [5]. At present, the main cultivation model of *P. notoginseng* is monoculture in field or greenhouses, imitating conventional crops [6]. The cultivation model in forest, with a high level of biodiversity, can supply a suitable growing environment that can use allelopathy to stimulate the plant’s growth and prevent pests and diseases [7,8]. Forest cultivation is a developing planting model that is used to improve the quality and safety of medicinal herbs [7,9,10]. Therefore, it is highly necessary to establish discrimination methods for *P. notoginseng* from different cultivation models.

Due to the presence of fraudulent practices of commodity identification, there is an urgent need to strengthen the testing and certification of foods, as well to use discriminatory science and technology for their regulation and verification, such as chemical analysis [11,12] and molecular detection [13,14]. A geographical indication (GI) has been introduced by farmers, manufacturers, or governments to protect agricultural products or foods, and the quality of food in a particular region is considered the best and most distinctive by consumers, especially in the field of herbal medicines [12,15,16]. At present, the origin traceability methods in food mainly include stable isotopes [17,18], multi-element composition [19,20], the characteristic content of organic components [21,22], near-infrared spectra [23,24], and high-throughput sequencing [25]. Among these, methods for origin tracing through taking advantage of multi-element are increasingly used because of their stability and accuracy. Multi-element composition can be determined in commodities, such as wine [26,27], tea [28,29], coffee [30], olive oil [31], and octopus [32], and is also used in the origin discrimination of *P. notoginseng* [33]. The methods used to determine content of elements in *P. notoginseng* include atomic absorption spectroscopy (AAS) [34], atomic fluorescence spectrometry (AFS) [35], inductively coupled plasma optical emission spectrometer (ICP-OES) [36], and inductively coupled plasma mass spectrometry (ICP-MS) [30]. However, these methods suffer from some common problems that hamper the rapid determination of multi-element composition in *P. notoginseng*, and affect their accuracy and traceability. In terms of multi-element determination, ICP-MS/MS reduces elemental interference [37] and has higher accuracy and greater diversity of elements compared to other methods [38,39].

In order to obtain higher accuracy and greater diversity of the content of multi-element in *P. notoginseng*, this study established the ICP-MS/MS method for 49 elements. Then, the accumulation dynamics of elements in *P. notoginseng* after transplanting were analyzed for guiding the addition of elements during the growth of *P. notoginseng.* Further, based on the content of multi-element in *P. notoginseng* samples (including five growing origins and two cultivation models), various modeling algorithms, such as partial least squares discriminant analysis (PLS-DA), logistic regression (LR), linear discriminant analysis (LDA), random forest (RF), the Naive Bayes algorithm (NB), k-nearest neighbors (kNNs), support vector machines (SVMs), and neural networks (NNs), were used to train and predict the discriminant model of origins and cultivation models for screening out the model that could determine origin and cultivation type accurately. This study could provide support for the transparency and traceability of supply chains of *P. notoginseng* and thus effectively avoid the occurrence of the fraud of geographical indications.

## 2. Results

### 2.1. Analytical Performance of the ICP-MS/MS for P. notoginseng

The procedure for the determination of multi-element in *P. notoginseng* samples by ICP-MS/MS was evaluated for its linearity, detection, and quantification limits (respectively, LODs and LOQs), as well as its accuracy and precision (Table S1, Supplementary Materials). The calibration curves for all the elements revealed a good linearity over the entire range of concentrations, with coefficients of determinations (R^2^) higher than 0.99, between 0.9919 and 0.9997. The instrumental LODs of ICP-MS/MS were between 0.0003 mg/kg (for ^200^Hg) and 7.716 mg/kg (for ^44^Ca); moreover, the LOQs were between 0.0011 mg/kg and 25.7202 mg/kg. The method proposed in this work showed good sensitivity for multi-element determination in *P. notoginseng* samples. The average recoveries of multi-element in *P. notoginseng* were in the range between 85.82% and 104.98% (Table S2); the relative standard deviation (RSDs) was in the range of 1.56%–9.70%, lower than 10%. Considering these results, it was concluded that this method had high accuracy and met the requirements of analyzing and measuring the content of multi-element in *P. notoginseng*.

### 2.2. The Accumulation Dynamics of Elements during the Growth of P. notoginseng

**Figure 1:**
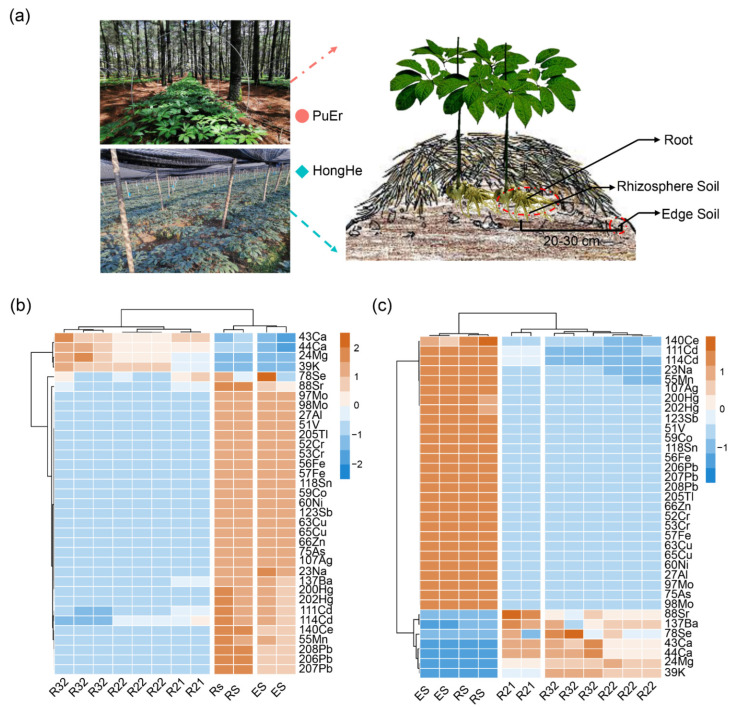
**The accumulation dynamics of elements during the growth of *P. notoginseng*.** (**a**) Growth environment of *P. notoginseng* in different cultivation models, and specific sampling locations of *P. notoginseng* roots, ehizosphere, and edge soil. (**b**) The multi-element determination result of *P. notoginseng* and soil in PuEr bases. (**c**) The multi-element determination result of *P. notoginseng* and soil in HongHe bases. RS: rhizosphere soil; ES: edge soil; R21: root of *P. notoginseng* collected in August 2019; R22: root of *P. notoginseng* collected in November 2019; and R32: root of *P. notoginseng* collected in November 2020.

The multi-element (total of twenty-six) determination of *P. notoginseng* (different time points in the same planting base) and soil (first sampling) was accomplished by the established ICP-MS/MS method. The heatmap of the multi-element content changes at different times (Figure 1b,c) showed that the contents of Ca, K, and Mg in *P. notoginseng* were significantly higher than those in soil, while the contents of other elements were lower than those in soil. With the growth extension, there was a trend of accumulation in the contents of Ca, K, and Mg in *P. notoginseng*, indicating a relatively large demand for these three elements during the growth process, which was consistent with the previous report [36].

### 2.3. Modeling Analysis of P. notoginseng from Different Origins

**Figure 2:**
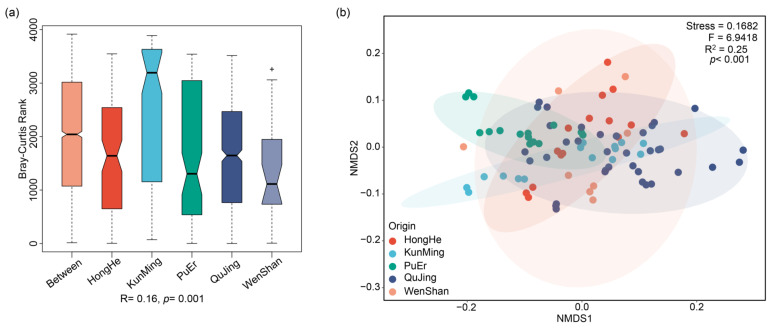
**The results of ANOSIM and NMDS analyses of multi-element content of *P. notoginseng* from five origins.** (**a**) The analysis of similarities (ANOSIM, using Bray–Curtis similarity distance matrix) of the multi-element content from five origins. ‘+’ represents outliers. (**b**) the results of the non-metric multidimensional scaling (NMDS, using the Bray–Curtis similarity distance matrix) of the multi-element content from five origins.

The multi-element determination of *P. notoginseng* samples, collected from different growing origins, was carried out by the established ICP-MS/MS method. By performing a Duncan’ test analysis on the multi-element determination results (Table S3), the content of ^23^Na, ^55^Mn, ^60^Ni, ^75^As, ^88^Sr, ^97^Mo, ^98^Mo, ^118^Sn, ^200^Hg, ^202^Hg, ^205^Tl, and ^232^Th had no significant difference between different origins (*p* > 0.05); the remaining 35 elements had significant differences between different origins. Based on the results of the analysis of similarities (ANOSIM, using Bray–Curtis similarity distance matrix) of the multi-element content from five origins (Figure 2a), R was 0.16 (*p* = 0.001), which showed that the differences in the content of multi-element from different origins were significantly greater than the differences between samples within the origin; thus, the grouping between different origins was reasonable. At the same time, the results of the non-metric multidimensional scaling (NMDS, using the Bray–Curtis similarity distance matrix) of the multi-element content from five origins (Figure 2b) (PERMANOVA analysis, F-value: 6.9418, R-squared: 0.25, *p*-value: < 0.001, stress: 0.1682) showed that there were significant differences in the content of multi-element in *P. notoginseng* from different origins, and the results of multi-element could be used to discriminate *P. notoginseng* from different origins.

There were seven machine learning algorithms, such as PLS-DA, LDA, RF, NB, kNNs, SVMs, and NNs, which were used to construct and evaluate the discriminative models of different origins based on the content of multi-element in *P. notoginseng*. The data were preprocessed, and the training and prediction sets were grouped (2:1). Then, the model was trained, blindly evaluated, and evaluated with the area under the ROC curve (AUC) (Table 1). The accuracy of NNs was 0.9259, the AUC value was 0.9750, and the *p*-value [Acc > NIR] was 2.32 × 10^−8^ < 0.0001, which were all significantly better than other algorithms. The sensitivity and specificity of five origins were also higher than other algorithms, indicating that the prediction model of origin discrimination by the NNs algorithm in this study could be applied to the discrimination of different origins of *P. notoginseng*.

### 2.4. Modeling Analysis of P. notoginseng from Different Cultivation Models

**Figure 3:**
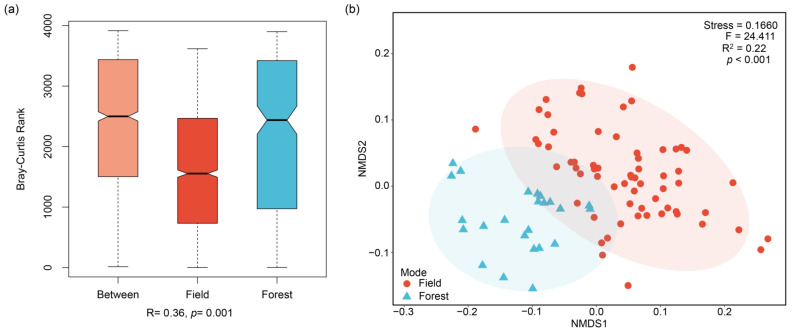
**The results of ANOSIM and NMDS analyses of the content of multi-element in *P. notoginseng* from different cultivation models.** (**a**) The analysis of similarities (ANOSIM, using Bray-Curtis similarity distance matrix) of the multi-element content from cultivation models. (**b**) the results of the non-metric multidimensional scaling (NMDS, using the Bray–Curtis similarity distance matrix) of the multi-element content from cultivation models.

The multi-element determination of *P. notoginseng*, collected from different cultivation models (field and forest), was accomplished by the established ICP-MS/MS method. After performing a T’ test analysis on the determination results of multi-element (Table S4), the contents of ^23^Na, ^60^Ni, ^63^Cu, ^65^Cu, ^88^Sr, ^97^Mo, ^98^Mo, ^118^Sn, ^153^Eu, ^200^Hg, ^202^Hg, ^205^Tl, and ^232^Th had no significant difference between the different models (ns). The contents of ^24^Mg, ^43^Ca, ^44^Ca, ^66^Zn, ^107^Ag, and ^137^Ba in *P. notoginseng* in the forest model were significantly higher than those in the field model. However, the remaining 30 elements had the opposite results. The results of the ANOSIM analysis (using the Bray–Curtis similarity distance matrix) of the content of multi-element in *P. notoginseng* from different cultivation models (Figure 3a), where R was 0.36 (*p* = 0.001), showed that the differences in the content of multi-elements from different cultivation models were significantly greater than the differences between samples within the model; thus, the grouping between different models was reasonable. NMDS analysis (using the Bray–Curtis similarity distance matrix) was carried out (Figure 3b) (PERMANOVA analysis, F-value: 24.411, R-squared: 0.22, *p*-value: < 0.001, stress: 0.1660), which showed that there were significant differences in the multi-element content of *P. notoginseng* from different models, and multi-element results could be used to discriminate *P. notoginseng* from different cultivation models.

There were eight machine learning algorithms, such as PLS-DA, LR, LDA, RF, NNs, kNNs, NB, and SVMs, which were used to construct and evaluate the discriminative models of different cultivation models based on the content of multi-element in *P. notoginseng*. The data were preprocessed, and the training and the prediction sets were grouped (7:3). Then, the model was trained, blindly evaluated, and evaluated with AUC (Table 2). The *p*-values [Acc > NIR] of PLS-DA, RF, and SVMs algorithms were <0.05, which showed that the prediction accuracies of these three models were significant. The accuracy and AUC of SVMs were both 1.0000 (best performance), indicating that the prediction model of cultivation model discrimination by the SVMs algorithm in this study could be applied to the discrimination of *P. notoginseng* in field or forest.

## 3. Discussion

In general, the content of multi-element in foods, especially agricultural products, may vary depending on factors, such as fertilizers, climatic conditions in the year of cultivation, differences in soil types, field history, and species in a single field [40], and is less affected by the processing process and storage time [41]. Therefore, multi-element can serve as a good geographical tracer as their distribution in the final product reflects the elemental signature in the soil of origin [36,42]. In this experiment, the ICP-MS/MS method was established to determine the content of 49 elements in *P. notoginseng* from different origins and cultivation models. Compared with the previous research methods [33,34,35,36], the ICP-MS/MS method in this work had good accuracy and sensitivity for multi-element determination in *P. notoginseng*. Moreover, the accumulation dynamics of multi-element of *P. notoginseng* after transplanting was analyzed, which showed that it had a relatively large demand for Ca, K, and Mg during the growth process, which may be due to the fact that Ca can ensure cell life activity, K can promote photosynthesis and increase plant resistance, and Mg is involved in plant photosynthesis and is an activator or component of many enzymes [43].

*P. notoginseng* has been used worldwide, is of significant economic value, is a significantly geographical indication product [33], and may have higher quality and safety when planted in forest [7,9]. Therefore, it is highly necessary to establish discrimination methods for *P. notoginseng* from different origins and cultivation models. In the field of data mining, many mistakes would be made throughout the analyses or attempting to establish relationships between multiple features. The chemometrics is a powerful tool in applying data mining, and thus can effectively solve the above problems [44,45], which can be divided into unsupervised algorithms and supervised algorithms. Among them, the supervised algorithms are used to classify samples into predefined classes, which is more helpful for the establishment of models [46,47]. In this study, the origin discriminant model using the NNs algorithm and the cultivation mode model using the SVMs algorithm were achieved based on the content of 49 elements in *P. notoginseng*. NNs, a series of algorithms that mimic the operations of a human brain to recognize relationships between vast amounts of data [48], was also used. At present, the study of geographical discrimination of edible oils [49], honey [50], French red wines [51], pork [52], and so on, had also proved that this algorithm could effectively help to establish the origin discrimination model. As one of the most popular supervised algorithms, SVMs was used to create the best line or decision boundary that can segregate n-dimensional space into classes; thus, easily put, the new data point toward the correct category in the future [53]. At present, the study of geographical discrimination of millet [54], *Curcumae Radix* [55], *Angelicae Sinensis Radix* [56], vegetables [57], and so on had also proved that this algorithm could effectively help to establish the discrimination model.

## 4. Material and Methods

### 4.1. Chemicals and Reagents

Nitric acid 65% (HNO_3_) was purchased from Merck, USA, and hydrofluoric acid 49% (HF) was purchased from Aladdin Reagent Corporation, China. Ultrapure deionized water (ddH_2_O) with a resistivity of 18.2 MΩ cm was obtained from a Milli-Q Plus water purification system (Millipore, Bedford, MA, USA).

Twenty-six multielement standard solutions (Na, Mg, K, Ca, Fe (1000 μg/mL), Sr (100 μg/mL), Al, V, Cr, Mn, Co, Ni, Cu, Zn, As, Se, Mo, Ag, Cd, Sb, Sn, Ba, Pb, Tl, Th, and U (10 μg/mL)), a single-element Hg standard solution, and seventeen rare-earth elements (Ce, Dy, Er, Eu, Gd, Ho, La, Lu, Nd, Pr, Sc, Sm, Tb, Th, Tm, Y, and Yb (10 μg/mL each)) were provided by Agilent Technologies Company.

### 4.2. Collection and Pretreatment of P. notoginseng

*P. notoginseng* samples (a total of 89 mixed samples) were collected from the main planting origins in Yunnan Province, China, in 2019 and 2020. The sampling points were shown in Table S5 (a total of 30 sampling bases), which included both the five main planting origins (WenShan, QuJing, HongHe, KunMing, and PuEr) and the cultivation model in field and forest of *P. notoginseng*. Each planting base was randomly sampled at three points, and eight or ten *P. notoginseng* samples were collected at each point as mixed samples. Then, the collected roots of *P. notoginseng* were washed with clean water, dried at 60 °C, coarsely crushed, ground to ultrafine powder with a Planetary Mono Mill (PULVERISETTE 6, Fritsch, Idar-Oberstein, Germany), and then passed through a 60 mesh sieve.

In addition, in order to study the accumulation dynamics of multi-element (not rare earth elements) in *P. notoginseng* after transplanting, two planting bases, PuEr (forest model) and HongHe (field model), were selected, and samples were collected in August 2019, November 2019, and November 2020, respectively. A three-point random sampling method was adopted for each base. Ten *P. notoginseng* plants were collected from each sampling point and 100 g of rhizosphere soil and edge soil was also collected during the first sampling. Then, samples from three points were mixed as the same treatment. The *P. notoginseng* samples were pretreated by the same method. Next, the soils were naturally dry and then passed through a 60 mesh sieve.

### 4.3. Microwave-Assisted Acid Digestion Procedure

All glassware and polytetrafluoroethylene (PTFE) tubes were immersed in a 10% (*v/v*) HNO_3_ solution for 48 h, followed by a minimum of three rinses with ddH_2_O, before being dried and finally stored ready for use [58]. A Multiwave PRO microwave digestion system (AntonPaar, Ashland, VA, USA) was used for the digestion of samples.

Soil: About 0.1 g of each soil sample was weighed and mixed with 4 mL of HNO_3_ and 2 mL of HF, and pre-digested at 130 °C for 30 min. The samples were then processed by microwave digestion with a ramped-up temperature from ambient to 130 °C over 10 min and held for 5 min, followed by a ramped-up temperature to 195 °C over 10 min and held for 20 min. After digestion, the solutions were evaporated to near dryness and cooled to room temperature. A negative control (no sample) was provided for each series of digestions.

*P. notoginseng*: About 0.4 g of each homogenized sample was weighed and mixed with 6 mL of HNO_3_, and pre-digested at 130 °C for 30 min. The samples were then processed by microwave digestion with a ramped-up temperature from ambient to 120 °C over 10 min and held for 2 min, followed by a ramped-up temperature to 190 °C over 4 min and held for 20 min. After digestion, the solutions were cooled to room temperature. A negative control (no sample) was provided for each series of digestions. Both digested samples and blanks were diluted to 50 mL with ddH_2_O and analyzed by ICP-MS/MS.

### 4.4. ICP-MS/MS Analysis

The concentration of 49 elements (^23^Na, ^24^Mg, ^27^Al, ^39^K, ^43^Ca, ^44^Ca, ^51^V, ^52^Cr, ^53^Cr, ^55^Mn, ^56^Fe, ^57^Fe, ^59^Co, ^60^Ni, ^63^Cu, ^65^Cu, ^66^Zn, ^75^As, ^78^Se, ^88^Sr, ^89^Y, ^97^Mo, ^98^Mo, ^107^Ag, ^111^Cd, ^114^Cd, ^118^Sn, ^123^Sb, ^137^Ba, ^139^La, ^140^Ce, ^141^Pr, ^146^Nd, ^147^Sm, ^153^Eu, ^157^Gd, ^163^Dy, ^165^Ho, ^166^Er, ^169^Tm, ^172^Yb, ^200^Hg, ^202^Hg, ^205^Tl, ^206^Pb, ^207^Pb, ^208^Pb, ^232^Th, and ^238^U) in *P. notoginseng* was determined by ICP-MS/MS (Agilent 8800, Tokyo, Japan). The calibration standard solutions were prepared in the range of 0–4 μg/mL for ^23^Na, ^24^Mg, ^39^K, ^43^Ca, ^44^Ca, ^56^Fe, and ^57^Fe; 0–400 ng/mL for ^27^Al, ^66^Zn, ^88^Sr, and ^118^Sn; 0–40 ng/mL for ^51^V, ^52^Cr, ^53^Cr, ^55^Mn, ^59^Co, ^60^Ni, ^63^Cu, ^65^Cu, ^75^As, ^78^Se, ^89^Y, ^97^Mo, ^98^Mo, ^107^Ag, ^111^Cd, ^114^Cd, ^123^Sb, ^137^Ba, ^146^Nd, ^147^Sm, ^153^Eu, ^157^Gd, ^163^Dy, ^166^Er, ^172^Yb, ^205^Tl, ^206^Pb, ^207^Pb, ^208^Pb, ^232^Th, and ^238^U; 0–12.5 ng/mL for ^139^La, ^140^Ce, ^141^Pr, ^165^Ho, and ^169^Tm; and 0–8 ng/mL for ^200^Hg and ^202^Hg. A mixed internal standard (ISTD) solution with a concentration of 50 ng/mL Sc, Ge, In, and Bi was used to correct changes in the sample uptake rate and plasma conditions for the ICP-MS/MS [59]. Through the tuning program, the operational mode of He and no gas mode were optimized. The instrument’s other conditions of ICP-MS/MS were shown in Table S6.

The multi-element calibration solutions were prepared at different concentration levels using 5% HNO_3_ media to match the sample matrix. By analyzing the experimental data, a linear fitting standard curve with the *X*-axis as the concentration point and the *Y*-axis as the response value was created. Using this standard curve, a background equivalent concentration of the analysis element was obtained by calculating the element standard deviation. LODs were calculated as (3 σ/k) and the LOQs were calculated as (10 σ/k), where standard deviation (σ) was the standard deviation of the blank signal (n = 11) and k was the slope of the calibration line [33,60]. Then, the accuracy of the method was estimated using analytical recovery, which was evaluated by adding the standard solutions with two different concentration levels (high and low) to *P. notoginseng* samples. These samples were both digested and analyzed in triplicate by ICP-MS/MS [22].

### 4.5. Statistical Analysis

All statistical analyses were conducted in the R software environment (v4.1.2; http://www.r-project.org/, accessed on 18 January 2022). Most of the results were visualized using the ‘ggplot2′ package [61], unless otherwise indicated. The experimental data were expressed as mean ± S.E.M, and recorded in Excel 2019 (Microsoft); then, the significance analysis was performed using the ‘agricolae’ package [62] and ‘ggpubr’ package [63] for Duncan’s test and T’test, respectively. The permutational multivariate analysis of variance (PERMANOVA), Anosim, and NMDS were performed using the ‘vegan’ package [64]. Heatmaps were illustrated based on Z-score-normalized relative abundance of taxa using the ‘pheatmap’ package [65]. Discriminative models for *P. notoginseng* were trained and predicted using the ‘Caret’ package [66]. In the field of data mining, supervised algorithms were used to classify samples into predefined classes. This was helpful for the establishment of models [47], such as PLS-DA, LR, LDA, RF, NB, kNNs, SVMs, and NNs.

## 5. Conclusions

The discriminant models established in this study could be used to support transparency and traceability of supply chains of *P. notoginseng* and thus avoid the fraud of geographic identification. This study contributes toward generalizing the multi-element analysis coupled with chemometrics as a promising tool for discriminating the origin of medicinal herbs and food, and provides technical support for the relevant research of the origin discrimination.

## Figures and Tables

**Table 1:** The major parameters of models of each class based on the content of multi-element in *P. notoginseng* from different origins.

Modes	Region	Sensitivity	Specificity	Balanced Accuracy	Accuracy	*p*-Value [Acc > NIR]	Kappa Value	Area under ROC Curve (AUC)
PLS-DA	HongHe	0.7500	0.8696	0.8098	0.4815	2.76 × 10^−1^	0.3844	0.8750
	KunMing	1.0000	0.5652	0.7862				
	PuEr	0.5000	0.9565	0.7283				
	QuJing	0.0910	1.0000	0.5455				
	WenShan	0.7500	1.0000	0.8750				
LDA	HongHe	0.2500	0.9565	0.6033	0.7037	1.78 × 10^−3^	0.6129	0.7881
	KunMing	0.7500	0.9565	0.8533				
	PuEr	1.0000	0.9565	0.9783				
	QuJing	0.6364	0.8750	0.7557				
	WenShan	1.0000	0.8696	0.9348				
RF	HongHe	0.7500	0.9565	0.8533	0.8889	2.89 × 10^−7^	0.8472	0.8750
	KunMing	0.7500	1.0000	0.8750				
	PuEr	0.7500	1.0000	0.8750				
	QuJing	1.0000	0.8750	0.9375				
	WenShan	1.0000	1.0000	1.0000				
NNs	HongHe	1.0000	0.9565	0.9783	0.9259	2.32 × 10^−8^	0.902	0.9750
	KunMing	0.7500	0.9565	0.8533				
	PuEr	1.0000	1.0000	1.0000				
	QuJing	0.9091	1.0000	0.9545				
	WenShan	1.0000	1.0000	1.0000				
kNNs	HongHe	1.0000	0.9565	0.9783	0.6667	5.82 × 10^−3^	0.595	0.8920
	KunMing	0.7500	0.8261	0.7880				
	PuEr	1.0000	0.9565	0.9783				
	QuJing	0.2727	1.0000	0.6364				
	WenShan	1.0000	0.8696	0.9348				
NB	HongHe	0.7500	0.9565	0.8533	0.7407	4.63 × 10^−4^	0.6655	0.9071
	KunMing	1.0000	0.8261	0.9130				
	PuEr	0.7500	1.0000	0.8750				
	QuJing	0.6364	0.9375	0.7869				
	WenShan	0.7500	0.9565	0.8533				
SVMs	HongHe	1.0000	0.9565	0.9783	0.8889	2.89 × 10^−7^	0.8548	0.9625
	KunMing	0.7500	0.9130	0.8315				
	PuEr	1.0000	1.0000	1.0000				
	QuJing	0.8182	1.0000	0.9091				
	WenShan	1.0000	1.0000	1.0000				

**Table 2:** The major parameters of models of each class based on the content of multi-element in *P. notoginseng* under different planting models.

Modes	Sensitivity	Specificity	Accuracy	*p*-Value [Acc > NIR]	Kappa Value	Area under ROC Curve (AUC)
PLS-DA	1.0000	0.8571	0.9615	0.0030	0.8976	0.9286
LR	0.7368	1.0000	0.8077	0.2605	0.8261	0.8684
LDA	0.6842	1.0000	0.8571	0.4258	0.5385	0.8421
RF	1.0000	0.8571	0.9615	0.0030	0.8976	0.9286
NNs	0.8947	0.8571	0.8846	0.0531	0.7194	0.8759
kNNs	1.0000	0.5714	0.0531	0.0531	0.6609	0.7857
NB	0.7368	1.0000	0.8077	0.26045	0.6012	0.8684
SVMs	1.0000	1.0000	1.0000	0.0003	1.0000	1.0000

## Data Availability

The data presented in this study are available in this article and supplementary material.

## Supplementary Materials

The following supporting information can be downloaded at: https://www.mdpi.com/article/10.3390/molecules27092982/s1. Table S1: Linear ranges, equations, correlation coefficients (R2), LODs, and LOQs of the ICP-MS/MS for the determination of multi-elements in *P. notoginseng*, Table S2: The spike recovery and reproducibility of *P. notoginseng* (n = 3), Table S3: Multi-element contents and comparison results using Duncan’test for *P.notoginseng* of different geographical origins (mg/kg), Table S4: Multi-element contents and comparison results using T’test for *P.notoginseng* of different cultivation models (mg/kg), Table S5: The allocation of sampling areas for the *P. notoginseng* in Yunnan province, China, Table S6: Agilent 8800 ICP-MS/MS operating parameters.

## Sample Availability

Samples of the compounds are not available from the authors.

## References

[1] Yue J., Zuo Z., Huang H., Wang Y. (2021). Application of Identification and Evaluation Techniques for Ethnobotanical Medicinal Plant of Genus *Panax*: A Review. Crit. Rev. Anal. Chem..

[2] Chen S., Wu Y., Qin X., Wen P., Liu J., Yang M. (2021). Global gene expression analysis using RNA-seq reveals the new roles of *Panax notoginseng* saponins in ischemic cardiomyocytes. J. Ethnopharmacol..

[3] Yuan Z., Liao Y., Tian G., Li H., Jia Y., Zhang H., Tan Z., Li X., Deng W., Liu K. (2011). *Panax notoginseng* saponins inhibit zymosan a induced atherosclerosis by suppressing integrin expression, FAK activation and NF-κB translocation. J. Ethnopharmacol..

[4] Tan M.-M., Chen M.-H., Han F., Wang J.W., Tu Y.X. (2021). Role of Bioactive Constituents of *Panax notoginseng* in the Modulation of Tumorigenesis: A Potential Review for the Treatment of Cancer. Front. Pharmacol..

[5] Liao P., Liu P., Wang Y., Huang C., Lan L., Yang Y., Cui X. (2018). Stereoscopic cultivation of *Panax notoginseng*: A new approach to overcome the continuous cropping obstacle. Ind. Crop. Prod..

[6] Wang Y.L., Cui X.M., Lan L., Chen W.D., Li R.B., Wang C.X., Yang X.Y., Liu D.H., Yang Y. (2015). Light and temperature and their effects on photosynthesis characteristics of stereoscopic cultivation in *Panax notoginseng*. China J. Chin. Mater. Med..

[7] Ye C., Fang H.Y., Liu H.J., Yang M., Zhu S.S. (2019). Current status of soil sickness research on *Panax notoginseng* in Yunnan, China. Allelopath. J..

[8] Wu H., Xia J., Qin X., Wu H., Zhang S., Zhao Y., Rensing C., Lin W. (2020). Underlying Mechanism of Wild Radix pseudostellariae in Tolerance to Disease Under the Natural Forest Cover. Front. Microbiol..

[9] Chen W., Balan P., Popovich D.G. (2020). Ginsenosides analysis of New Zealand-grown forest *Panax ginseng* by LC-QTOF-MS/MS. J. Ginseng Res..

[10] Zhu L., Xu L., Dou D., Huang L. (2021). The distinct of chemical profiles of mountainous forest cultivated ginseng and garden ginseng based on ginsenosides and oligosaccharides. J. Food Compos. Anal..

[11] Ma G., Zhang Y., Zhang J., Wang G., Chen L., Zhang M., Liu T., Liu X., Lu C. (2016). Determining the geographical origin of Chinese green tea by linear discriminant analysis of trace metals and rare earth elements: Taking Dongting Biluochun as an example. Food Control.

[12] Lu Y., Yao G., Wang X., Zhang Y., Zhao J., Yu Y.-J., Wang H. (2022). Chemometric discrimination of the geographical origin of licorice in China by untargeted metabolomics. Food Chem..

[13] Wu Y.H., Dong Y., Shi Y.C., Yang H., Zhang J.Q., Khan M.R., Deng S., He G.P., He Q., Lv Y.P. (2021). CRISPR-Cas12-Based Rapid Authentication of Halal Food. J. Agric. Food Chem..

[14] Wu Y.H., Liu J., Li H.T., Zhang T., Dong Y., Deng S., Lv Y.P., He Q., Deng R.J. (2022). CRISPR-Cas sys-tem meets DNA barcoding: Development of a universal nucleic acid test for food authentication. Sens. Actuators B Chem..

[15] Liu G., Zhang Q., Yin G., Musyimi Z. (2016). Spatial distribution of geographical indications for agricultural products and their drivers in China. Environ. Earth. Sci..

[16] Ndraha N., Hsiao H.-I., Chih Wang W.C. (2017). Comparative study of imported food control systems of Tai-wan, Japan, the United States, and the European Union. Food Control.

[17] Wang J., Zhang T., Ge Y. (2021). C/N/H/O stable isotope analysis for determining the geographical origin of American ginseng (*Panax quinquefolius*). J. Food Compos. Anal..

[18] Su Y.-Y., Gao J., Zhao Y.-F., Wen H.-S., Zhang J.-J., Zhang A., Yuan C.-L. (2020). Geographical Origin Classification of Chinese Wines Based on Carbon and Oxygen Stable Isotopes and Elemental Profiles. J. Food Prot..

[19] Qian L., Zuo F., Liu H., Zhang C., Chi X., Zhang D. (2019). Determination of Geographical Origin of Wuchang Rice with the Geographical Indicator by Multielement Analysis. J. Food Qual..

[20] Ng W.L., Bay L.J., Goh G., Ang T.H., Kong K., Chew P., Koh S.P., Ch’ng A.L., Phang H., Chiew P. (2021). Multivariate statistical analysis of stable isotope signatures and element concentrations to differentiate the geographical origin of retail milk sold in Singapore. Food Control.

[21] Zhang J., Tian Z., Ma Y., Shao F., Huang J., Wu H., Tian L. (2019). Origin Identification of the Sauce-Flavor Chinese Baijiu by Organic Acids, Trace Elements, and the Stable Carbon Isotope Ratio. J. Food Qual..

[22] Du M., Fang Y., Shen F., Mao B., Zou Y., Li P., Pei F., Hu Q. (2018). Multiangle discrimination of geographical origin of rice based on analysis of mineral elements and characteristic volatile components. Int. J. Food Sci. Technol..

[23] Chen H., Tan C., Li H. (2021). Discrimination between wild-grown and cultivated Gastrodia elata by near-infrared spectroscopy and chemometrics. Vib. Spectrosc..

[24] Arndt M., Drees A., Ahlers C., Fischer M. (2020). Determination of the Geographical Origin of Walnuts (*Juglans regia* L.) Using Near-Infrared Spectroscopy and Chemometrics. Foods.

[25] Gao F., Zeng G., Wang B., Xiao J., Zhang L., Cheng W., Wang H., Li H., Shi X. (2021). Discrimination of the geographic origins and varieties of wine grapes using high-throughput sequencing assisted by a random forest model. LWT.

[26] Orellana S., Johansen A.M., Gazis C. (2019). Geographic classification of U.S. Washington State wines using elemental and water isotope composition. Food Chem. X.

[27] Leder R., Petric I.V., Jusup J., Banović M. (2021). Geographical Discrimination of Croatian Wines by Stable Isotope Ratios and Multielemental Composition Analysis. Front. Nutr..

[28] Wang Y., Kang L., Zhao Y., Xiong F., Yuan Y., Nie J., Huang L., Yang J. (2022). Stable isotope and multi-element profiling of Cassiae Semen tea combined with chemometrics for geographical discrimination. J. Food Compos. Anal..

[29] Zhang M., Huang C., Zhang J., Qin H., Ma G., Liu X., Yin J. (2020). Accurate discrimination of tea from multiple geographical regions by combining multi-elements with multivariate statistical analysis. J. Food Meas. Charaterization.

[30] Endaye M., Atlabachew M., Mehari B., Alemayehu M., Mengistu D.A., Kerisew B. (2020). Combining Multi-Element Analysis with Statistical Modeling for Tracing the Origin of Green Coffee Beans from Amhara Region, Ethiopia. Biol. Trace Elem. Res..

[31] Beltrán M., Sánchez-Astudillo M., Aparicio R., García-González D.L. (2015). Geographical traceability of virgin olive oils from south-western Spain by their multi-elemental composition. Food Chem..

[32] Martino J.C., Mazumder D., Gadd P., Doubleday Z.A. (2022). Tracking the provenance of octopus using isotopic and multi-elemental analysis. Food Chem..

[33] Lv H., Zhang Y., Sun Y., Duan Y. (2019). Elemental characteristics of Sanqi (*Panax notoginseng*) in Yunnan province of China: Multielement determination by ICP-AES and ICP-MS and statistical analysis. Microchem. J..

[34] Yin Q., Zhu Y., Ju S., Liao W., Yang Y. (2016). Rapid determination of copper and lead in *Panax notoginseng* by magnetic solid-phase extraction and flame atomic absorption spectrometry. Res. Chem. Intermed..

[35] Pan Q., Liu W., Yang J., Min Y., Huang Z. (2013). Determination of Arsenic in *Panax notoginseng* by Hydride Generation Atomic Fluorescence Spectrometry. Asian J. Chem..

[36] Zheng Y., Xia P., Chai W., Liang Z., Yan K. (2020). Accumulation dynamics of elements in *Panax notoginseng* during its whole growing seasons. Ind. Crop. Prod..

[37] Leyden E., Farkas J., Gilbert S., Hutson J., Mosley L.M. (2021). A simple and rapid ICP-MS/MS determination of sulfur isotope ratios (34S/32S) in complex natural waters: A new tool for tracing seawater intrusion in coastal systems. Talanta.

[38] Hirata J., Itabashi D., Aimoto M. (2021). Determination of Ultra-trace Tellurium in Steel by ID-ICP-MS/MS with Liquid-Liquid Extraction. Anal. Sci..

[39] Simpson A., Gilbert S., Tamblyn R., Hand M., Spandler C., Gillespie J., Nixon A., Glorie S. (2021). In Situ Lu Hf geochronology of garnet, apatite and xenotime by LA ICP MS/MS. Chem. Geol..

[40] Ariyama K., Nishida T., Noda T., Kadokura M., Yasui A. (2006). Effects of fertilization, crop year, variety, and provenance factors on mineral concentrations in onions. J. Agric. Food Chem..

[41] Zhao H., Zhang S., Zhang Z. (2017). Relationship between multi-element composition in tea leaves and in provenance soils for geographical traceability. Food Control.

[42] Carini F., Bengtsson G. (2001). Post-deposition transport of radionuclides in fruit. J. Environ. Radioact..

[43] Wang X., Li L., Zhang S.C. (2019). Plant Physiology.

[44] Kotsiantis S.B., Zaharakis I.D., Pintelas P.E. (2006). Machine learning: A review of classification and combining techniques. Artif. Intell. Rev..

[45] Yuan Y., Hu G., Chen T., Zhao M., Zhang Y., Li Y., Xu X., Shao S., Zhu J., Wang Q. (2016). Improved Discrimination for Brassica Vegetables Treated with Agricultural Fertilizers Using a Combined Chemometric Approach. J. Agric. Food Chem..

[46] Liu H.-L., Zeng Y.-T., Zhao X., Tong H.-R. (2020). Improved geographical origin discrimination for tea using ICP-MS and ICP-OES techniques in combination with chemometric approach. J. Sci. Food Agric..

[47] Wold S., Ruhe A., Wold H., Dunn W.J. (1984). The Collinearity Problem in Linear Regression. The Partial Least Squares (PLS) Approach to Generalized Inverses. SIAM J. Sci. Stat. Comput..

[48] Schmidhuber J. (2015). Deep learning in neural networks: An overview. Neural Netw..

[49] Liu Y., Yao L., Xia Z., Gao Y., Gong Z. (2021). Geographical discrimination and adulteration analysis for edible oils using two-dimensional correlation spectroscopy and convolutional neural networks (CNNs). Spectrochim. Acta Part A Mol. Biomol. Spectrosc..

[50] Drivelos S.A., Danezis G.P., Halagarda M., Popek S., Georgiou C.A. (2021). Geographical origin and botanical type honey authentication through elemental metabolomics via chemometrics. Food Chem..

[51] Wu H., Lin G., Tian L., Yan Z., Yi B., Bian X., Jin B., Xie L., Zhou H., Rogers K.M. (2021). Origin verification of French red wines using isotope and elemental analyses coupled with chemometrics. Food Chem..

[52] Qi J., Li Y., Zhang C., Wang C., Wang J., Guo W., Wang S. (2021). Geographic origin discrimination of pork from different Chinese regions using mineral elements analysis assisted by machine learning techniques. Food Chem..

[53] Boser B.E., Guyon I.M., Vapnik V.N., Haussler D. (1992). A Training Algorithm for Optimal Margin Classifiers. Proceedings of the COLT 92: The 5th Annual Workshop on Computational Learning Theory.

[54] Kabir M.H., Guindo M.L., Chen R., Liu F. (2021). Geographic Origin Discrimination of Millet Using Vis-NIR Spectroscopy Combined with Machine Learning Techniques. Foods.

[55] Wang L., Wang X., Liu X., Wang Y., Ren X., Dong Y., Song R., Ma J., Fan Q., Wei J. (2021). Fast discrimination and quantification analysis of Curcumae Radix from four botanical origins using NIR spectroscopy coupled with chemometrics tools. Spectrochim. Acta Part A Mol. Biomol. Spectrosc..

[56] Zhang Z.-Y., Wang Y.-J., Yan H., Chang X.-W., Zhou G.-S., Zhu L., Liu P., Guo S., Dong T.T.X., Duan J.-A. (2021). Rapid Geographical Origin Identification and Quality Assessment of Angelicae Sinensis Radix by FT-NIR Spectroscopy. J. Anal. Methods Chem..

[57] Liu X., Liu Z., Qian Q., Song W., Rogers K.M., Rao Q., Wang S., Zhang Q., Shao S., Tian M.L. (2021). Isotope chemometrics determines farming methods and geographical origin of vegetables from Yangtze River Delta Region, China. Food Chem..

[58] Nasr E.G., Epova E.N., de Diego A., Souissi R., Hammami M., Abderrazak H., Donard O.F.X. (2021). Trace Elements Analysis of Tunisian and European Extra Virgin Olive Oils by ICP-MS and Chemometrics for Geographical Discrimination. Foods.

[59] Nguyen-Quang T., Bui-Quang M., Truong-Ngoc M. (2021). Rapid Identification of Geographical Origin of Commercial Soybean Marketed in Vietnam by ICP-MS. J. Anal. Methods Chem..

[60] de Souza M.J., Barciela-Alonso M.C., Aboal-Somoza M., Bermejo-Barrera P. (2021). Determination of the Trace Element Contents of Fruit Juice Samples by ICP OES and ICP-MS. Braz. J. Anal. Chem..

[61] Gómez-Rubio V. (2017). *ggplot2-Elegant Graphics for Data Analysis*, 2nd ed. ; J. Stat. Soft..

[62] de Mendiburu F. Agricolae: Statistical Procedures for Agricultural Research. https://cran.r-project.org/web/packages/agricolae/agricolae.pdf.

[63] Kassambara A. ggpubr R Package: ggplot2-Based Publication Ready Plots. https://rpkgs.datanovia.com/ggpubr.

[64] Dixon P. (2003). VEGAN, a package of R functions for community ecology. J. Veg. Sci..

[65] Kolde R. Pheatmap: Pretty Heatmaps. https://rdrr.io/cran/pheatmap/.

[66] Kumar A. (2018). Pre-processing and Modelling using Caret Package in R. Int. J. Comput. Appl..

