# Supplementary material for: Multi-Element Analysis and Origin Discrimination of Panax notoginseng Based on Inductively Coupled Plasma Tandem Mass Spectrometry (ICP-MS/MS)
Source: Molecules. 2022 May 6;27(9):2982. doi: 10.3390/molecules27092982 (PMC9105934; doi:10.3390/molecules27092982)
Supplement: Supplementary file 1 [file molecules-27-02982-s001.zip › molecules-1700137-supplementary.pdf]

Supplementary Materials

Multi-Element Analysis and Origin Discrimination of *Panax notoginseng* Based on Inductively Coupled Plasma Tandem Mass Spectrometry (ICP-MS/MS)

Chao Ji [1,†], Jinyu Liu [1,†], Qin Zhang [2], Juan Li [2], Zhiqiang Wu [2], Xingyu Wang [2], Yuxin Xie [2], Jiangchao Zhao [3], Rui Shi [4], Xing Ma [5], Mohammad Rizwan Khan [6], Rosa Busquets [7], Xiahong He [1,4], Youyong Zhu [1], Shusheng Zhu [1,*] and Wenjie Zheng [1,2,4,*]

[1] State Key Laboratory for Conservation and Utilization of Bio-Resources in Yunnan, National Engineering Research Center for Applied Technology of Agricultural Biodiversity, College of Plant Protection, Yunnan Agricultural University, Kunming 650201, China; jichao1100@163.com (C.J.); dianchahuyihao@163.com (J.L.); hexiahong@hotmail.com (X.H.); yyzhu@ynau.edu.cn (Y.Z.)

[2] Laboratory for Quality Control and Traceability of Food and Agricultural Products, Tianjin Normal University, Tianjin 300387, China; 17854265918@163.com (Q.Z.); lijuandk0916@163.com (J.L.); wuzhiqiang314@126.com (Z.W.); wangxingyu100198@163.com (X.W.); wugeheshimo@163.com (Y.X.)

[3] Department of Animal Science, Division of Agriculture, University of Arkansas, Fayetteville, AR 72701, USA; jzhao77@uark.edu

[4] Key Laboratory for Forest Resources Conservation and Utilization in the Southwest Mountains of China, Ministry of Education, Southwest Landscape Architecture Engineering Research Center of National Forestry and Grassland Administration, Southwest Forestry University, Kunming 650224, China; shirui@swfu.edu.cn

[5] The Animal, Plant & Foodstuff Inspection Center, Tianjin Customs, Tianjin 300387, China; xingma2005@126.com

[6] Department of Chemistry, College of Science, King Saud University, Riyadh 11451, Saudi Arabia; mrkhan@ksu.edu.sa

[7] School of Life Sciences, Pharmacy and Chemistry, Kingston University London, Penrhyn Road, KT1 2EE, Kingston Upon Thames, UK; r.busquets@kingston.ac.uk

* Correspondence: shushengzhu79@126.com (S.Z.); skyzwj@tjnu.edu.cn (W.Z.)

† These authors contributed equally to this work.

Table S1. Linear ranges, equations, correlation coefficients (R^2), LODs, and LOQs of the ICP-MS/MS for the determination of multi-elements in *P. notoginseng*.

Element	Linear Range (ng/mL)	Equation	R^2	LODs (mg/kg)	LOQs (mg/kg)
^{23}Na	6.62-4000	y = 155.08x + 15060	0.9994	0.2484	0.8280
^{24}Mg	7.93-4000	y = 59.433x + 375.77	0.9995	0.2975	0.9916
^{27}Al	5.22-400	y = 13.565x + 79.102	0.9992	0.1956	0.6520
^{39}K	32.00-4000	y = 19.893x + 4234.7	0.9996	1.2000	4.0001
^{43}Ca	110.89-4000	y = 0.2365x + 50.829	0.9926	4.1585	13.8618
^{44}Ca	205.76-4000	y = 2.2262x + 284.74	0.9989	7.7160	25.7202
^{51}V	0.09-40	y = 578.88x + 23.959	0.9996	0.0035	0.0115
^{52}Cr	0.24-40	y = 785.24x + 386.76	0.9993	0.0088	0.0295
^{53}Cr	0.20-40	y = 98.633x + 61.657	0.9987	0.0073	0.0244
^{55}Mn	0.16-40	y = 252.05x + 169.71	0.9994	0.0058	0.0195
^{56}Fe	5.49-4000	y = 699.73x - 46446	0.9989	0.2059	0.6863
^{57}Fe	8.56-4000	y = 14.033x + 18.482	0.9995	0.3212	1.0706
^{59}Co	0.15-40	y = 1422.6x + 313.2	0.9993	0.0056	0.0185
^{60}Ni	0.35-40	y = 402.28x + 367.71	0.9994	0.0131	0.0438
^{63}Cu	1.25-40	y = 1061.6x + 1238.9	0.9992	0.0468	0.1559
^{65}Cu	0.94-40	y = 531.46x + 648.87	0.9993	0.0354	0.1181
^{66}Zn	2.54-400	y = 126.07x + 400.91	0.9994	0.0954	0.3179
^{75}As	0.08-40	y = 77.049x + 23.903	0.9987	0.0031	0.0104
^{78}Se	0.22-40	y = 7.9761x - 1.3532	0.9987	0.0083	0.0275
^{88}Sr	0.97-400	y = 180.8x + 634.56	0.9992	0.0362	0.1208
^{89}Y	0.02-40	y = 30488x - 40468	0.9919	0.0006	0.0022
^{97}Mo	0.07-40	y = 323.98x + 61.331	0.9995	0.0026	0.0088
^{98}Mo	0.05-40	y = 867.47x + 31.601	0.9997	0.0018	0.0059
^{107}Ag	0.08-40	y = 935.77x + 35.634	0.9995	0.0031	0.0103
^{111}Cd	0.09-40	y = 171.63x + 2.9439	0.9997	0.0033	0.0109
^{114}Cd	0.10-40	y = 438.28x + 65.226	0.9995	0.0037	0.0122
^{118}Sn	0.64-400	y = 25.977x + 29.411	0.9996	0.0241	0.0802
^{123}Sb	0.08-40	y = 284.63x + 20.599	0.9991	0.0030	0.0102
^{137}Ba	0.71-40	y = 97.506x + 55.9	0.9987	0.0267	0.0890
^{139}La	0.07-12.5	y = 34256x - 6346.8	0.9993	0.0028	0.0093
^{140}Ce	0.09-12.5	y = 31855x - 4472.6	0.999	0.0033	0.0111
^{141}Pr	0.04-12.5	y = 35858x - 4611.6	0.9991	0.0014	0.0045
^{146}Nd	0.06-40	y = 3677.4x - 136.72	0.9995	0.0024	0.0079
^{147}Sm	0.05-40	y = 3048.4x - 324.62	0.9997	0.0019	0.0062
^{153}Eu	0.01-40	y = 10360x - 1141.9	0.9995	0.0005	0.0016
^{157}Gd	0.03-40	y = 3300.1x + 119.2	0.9992	0.0010	0.0034

Element	Linear Range (ng/mL)	Equation	R^2	LODs (mg/kg)	LOQs (mg/kg)
^{163}Dy	0.01-40	y = 4476.5x - 458.3	0.9996	0.0005	0.0016
^{165}Ho	0.03-12.5	y = 28814x - 5256.3	0.9995	0.0011	0.0037
^{166}Er	0.01-40	y = 5846.5x - 597.19	0.9996	0.0005	0.0015
^{169}Tm	0.03-12.5	y = 28662x - 5784.1	0.9994	0.0012	0.0038
^{172}Yb	0.01-40	y = 3743.8x - 668.1	0.9996	0.0004	0.0013
^{200}Hg	0.01-8	y = 2359.2x + 38.878	0.9995	0.0003	0.0011
^{202}Hg	0.01-8	y = 3119.2x + 10.011	0.9995	0.0004	0.0012
^{205}Tl	0.02-40	y = 3232.2x + 820.02	0.9991	0.0008	0.0027
^{206}Pb	0.12-40	y = 1052.3x + 274.81	0.9992	0.0043	0.0144
^{207}Pb	0.09-40	y = 922.91x + 192.78	0.9993	0.0032	0.0108
^{208}Pb	0.11-40	y = 4246.2x + 1077.5	0.9992	0.0043	0.0143
^{232}Th	0.03-40	y = 7967.5x + 50.934	0.9994	0.0010	0.0034
^{238}U	0.02-40	y = 4664.6x + 1836.9	0.9989	0.0009	0.0028

Table S2. The spike recovery and reproducibility of *P. notoginseng* (n= 3).

Element	Background (mg/kg)	Added (mg/kg)	Recovery (%)	RSD (%)
^{23}Na	31.21	200	99.56	1.87
		20	100.62	4.29
^{24}Mg	2453.25	2000	96.12	7.60
		200	95.04	9.35
^{27}Al	232.26	200	87.04	5.93
		20	93.70	6.66
^{39}K	28495.69	2000	94.77	3.63
		200	95.05	9.14
^{43}Ca	1064.43	2000	97.00	3.54
		200	91.95	5.54
^{44}Ca	1491.75	2000	98.60	3.56
		200	86.79	3.20
^{51}V	0.40	20	99.45	4.27
		5	92.97	7.08
^{52}Cr	0.54	20	97.16	4.66
		5	97.09	5.67
^{53}Cr	0.60	20	99.20	6.55
		5	96.72	5.01
^{55}Mn	67.92	20	90.54	4.35
		5	99.45	9.31
^{56}Fe	51.17	20	96.99	3.46
		5	92.93	3.97
^{57}Fe	70.49	20	94.41	9.40
		5	86.75	4.01
^{59}Co	0.12	2	96.02	4.44
		0.5	96.73	7.88
^{60}Ni	2.64	2	95.88	7.56
		0.5	96.20	9.63
^{63}Cu	5.23	20	94.57	2.48
		5	101.62	4.41
^{65}Cu	5.27	20	99.13	3.58
		5	93.20	8.66
^{66}Zn	14.50	20	95.83	7.37
		5	88.66	3.83
^{75}As	0.14	2	95.71	4.06
		0.5	93.51	1.67
^{78}Se	0.12	2	93.35	2.09
		0.5	96.05	6.83

Element	Background (mg/kg)	Added (mg/kg)	Recovery (%)	RSD (%)
^{88}Sr	5.53	20	95.66	5.92
		5	97.31	4.99
^{89}Y	0.71	2	95.31	7.95
		0.5	91.90	5.01
^{97}Mo	0.02	2	97.38	8.02
		0.5	96.19	1.56
^{98}Mo	0.02	2	100.75	3.11
		0.5	104.98	2.42
^{107}Ag	0.01	2	98.76	3.90
		0.5	98.36	4.22
^{111}Cd	0.54	2	94.47	8.35
		0.5	94.55	5.05
^{114}Cd	0.54	2	95.36	5.99
		0.5	94.78	9.26
^{118}Sn	0.05	2	100.91	2.77
		0.5	89.62	5.79
^{123}Sb	0.07	2	96.62	9.38
		0.5	85.82	2.17
^{137}Ba	17.64	2	95.12	8.59
		0.5	93.74	9.70
^{139}La	0.57	2	96.66	8.42
		0.5	98.66	9.47
^{140}Ce	0.34	2	95.61	7.05
		0.5	97.79	5.30
^{141}Pr	0.08	2	95.78	4.55
		0.5	88.46	5.69
^{146}Nd	0.57	2	91.72	5.63
		0.5	98.87	9.63
^{147}Sm	0.10	2	92.45	3.35
		0.5	97.11	5.79
^{153}Eu	0.03	2	97.71	3.71
		0.5	96.86	3.76
^{157}Gd	0.12	2	98.85	7.58
		0.5	95.41	5.70
^{163}Dy	0.09	2	93.64	2.09
		0.5	95.21	8.29
^{165}Ho	0.01	2	100.47	3.71
		0.5	98.53	2.99
^{166}Er	0.06	2	98.04	2.02

Element	Background (mg/kg)	Added (mg/kg)	Recovery (%)	RSD (%)
		0.5	91.51	8.31
^{169}Tm	0.003	2	98.33	7.80
		0.5	99.97	2.97
^{172}Yb	0.04	2	97.24	4.62
		0.5	88.31	6.40
^{200}Hg	0.003	2	100.84	2.59
		0.5	102.70	6.00
^{202}Hg	0.003	2	100.86	2.61
		0.5	102.75	5.95
^{205}Tl	0.02	2	99.78	5.19
		0.5	100.44	5.30
^{206}Pb	0.53	2	95.17	8.20
		0.5	97.34	4.76
^{207}Pb	0.53	2	95.26	8.25
		0.5	97.73	5.93
^{208}Pb	0.53	2	95.00	8.47
		0.5	96.65	6.14
^{232}Th	0.01	2	100.73	2.69
		0.5	98.93	2.43
^{238}U	0.005	2	96.42	6.05
		0.5	95.02	5.63

Table S3. Multi-element contents and comparison results using Duncan'test for *P.notoginseng* of different geographical origins (mg/kg). Different uppercase and lowercase letters indicate significant differences between origins (P＜0.05).

Elements	HongHe (n=15)	KunMing (n=15)	PuEr (n=15)	QuJing (n=36)	WenShan (n=8)	F-value	P-value
^{23}Na	25.41 ± 2.88a	19.64 ± 2.47a	23.86 ± 4.66a	26.78 ± 3.77a	22.74 ± 2.69a	1.457	0.223
^{24}Mg	1791.29 ± 134.49bc	1513.98 ± 156.71c	2265.59 ± 181.24a	1501.54 ± 127.61c	1843.46 ± 149.06b	10.33	0.000
^{27}Al	425.63 ± 58.51b	558.28 ± 94.08ab	323.5 ± 29.99b	669.35 ± 62.95a	377.73 ± 72.88b	4.424	0.003
^{39}K	24682.91 ± 757.19a	18585.65 ± 1281.09c	21590.17 ± 843.64b	24082.02 ± 482.13ab	24437.46 ± 876.67a	9.318	0.000
^{43}Ca	979.63 ± 66.21bc	955.17 ± 71.16c	1232.66 ± 61.6a	958.34 ± 24.57c	1124.39 ± 43.09ab	5.699	0.000
^{44}Ca	1353.93 ± 89.79b	1333.55 ± 99.4b	1729.79 ± 86.82a	1304.64 ± 34b	1570.86 ± 67.3a	6.764	0.000
^{51}V	1.3 ± 0.27ab	1.25 ± 0.2ab	0.58 ± 0.07b	1.7 ± 0.18a	1.14 ± 0.27ab	4.17	0.004
^{52}Cr	1.49 ± 0.28ab	1.38 ± 0.21ab	0.79 ± 0.1b	2.18 ± 0.34a	0.76 ± 0.2b	3.433	0.012
^{53}Cr	1.38 ± 0.26ab	1.32 ± 0.2ab	0.75 ± 0.09b	2.09 ± 0.32a	0.77 ± 0.2b	3.405	0.013
^{55}Mn	49.28 ± 3.97ab	57.57 ± 5.21a	40.27 ± 2.96b	41.75 ± 3.85b	40.52 ± 6.53b	2.313	0.064
^{56}Fe	263.33 ± 67.07b	264.33 ± 47.88b	115.29 ± 15.16b	493.33 ± 69.36a	226.21 ± 76.52b	4.991	0.001
^{57}Fe	262.74 ± 63.4ab	260.59 ± 43.33ab	135.85 ± 12.5b	477.91 ± 67.41a	223.87 ± 68.86b	4.57	0.002
^{59}Co	0.33 ± 0.04ab	0.23 ± 0.03bc	0.11 ± 0.01c	0.47 ± 0.05a	0.26 ± 0.02bc	9.456	0.000
^{60}Ni	1.73 ± 0.14b	2.53 ± 0.32a	2.01 ± 0.11ab	2 ± 0.11ab	1.93 ± 0.52ab	1.872	0.123
^{63}Cu	3.48 ± 0.23b	3.39 ± 0.22b	4.55 ± 0.29a	3.87 ± 0.18ab	4.64 ± 0.47a	3.98	0.005
^{65}Cu	3.43 ± 0.31b	3.39 ± 0.3b	4.56 ± 0.4a	3.78 ± 0.37b	4.68 ± 0.5a	4.434	0.003
^{66}Zn	11.16 ± 0.8c	19.16 ± 3.28a	17.8 ± 2.18ab	14.32 ± 1.69bc	15.44 ± 1.5abc	4.673	0.002
^{75}As	0.46 ± 0.18a	0.26 ± 0.06ab	0.18 ± 0.03b	0.33 ± 0.08ab	0.28 ± 0.07ab	2.154	0.081
^{78}Se	0.03 ± 0.01ab	0.02 ± 0.01b	0.03 ± 0.01ab	0.05 ± 0.01a	0.04 ± 0.005ab	3.268	0.015

Elements	HongHe (n=15)	KunMing (n=15)	PuEr (n=15)	QuJing (n=36)	WenShan (n=8)	F-value	P-value
^{88}Sr	7.86 ± 1.55a	5.99 ± 0.81a	8.24 ± 1.87a	6.56 ± 0.47a	6.26 ± 0.46a	1.571	0.190
^{89}Y	0.11 ± 0.01ab	0.09 ± 0.01b	0.12 ± 0.01ab	0.19 ± 0.02a	0.16 ± 0.04ab	3.806	0.007
^{97}Mo	0.09 ± 0.06ab	0.04 ± 0.01b	0.08 ± 0.01b	0.06 ± 0.01b	0.16 ± 0.08a	2.018	0.099
^{98}Mo	0.09 ± 0.06ab	0.04 ± 0.01b	0.07 ± 0.01b	0.06 ± 0.01b	0.16 ± 0.08a	1.955	0.109
^{107}Ag	0.004 ± 0.001b	0.006 ± 0.001b	0.037 ± 0.016a	0.005 ± 0.001b	0.004 ± 0.001b	9.284	0.000
^{111}Cd	0.26 ± 0.05b	0.81 ± 0.17a	0.35 ± 0.14b	0.46 ± 0.08b	0.34 ± 0.13b	6.705	0.000
^{114}Cd	0.25 ± 0.05b	0.8 ± 0.17a	0.35 ± 0.14b	0.45 ± 0.08b	0.33 ± 0.13b	7.023	0.000
^{118}Sn	0.07 ± 0.03ab	0.12 ± 0.03ab	0.07 ± 0.01ab	0.15 ± 0.06a	0.02 ± 0.01b	2.443	0.053
^{123}Sb	0.18 ± 0.05a	0.08 ± 0.01b	0.07 ± 0.01b	0.09 ± 0.02b	0.11 ± 0.01b	6.586	0.000
^{137}Ba	13.38 ± 0.97c	19.4 ± 4b	24.73 ± 2.84a	15.09 ± 0.98bc	16.34 ± 2.2bc	8.214	0.000
^{139}La	0.17 ± 0.02b	0.2 ± 0.03ab	0.16 ± 0.02b	0.29 ± 0.08a	0.3 ± 0.06a	3.268	0.015
^{140}Ce	0.29 ± 0.03bc	0.39 ± 0.05abc	0.19 ± 0.03c	0.59 ± 0.07a	0.52 ± 0.1ab	5.839	0.000
^{141}Pr	0.03 ± 0.004b	0.03 ± 0.01b	0.03 ± 0.003b	0.06 ± 0.01a	0.05 ± 0.01ab	5.351	0.001
^{146}Nd	0.19 ± 0.03b	0.21 ± 0.03b	0.16 ± 0.02b	0.35 ± 0.09a	0.27 ± 0.04ab	4.594	0.002
^{147}Sm	0.04 ± 0.01b	0.04 ± 0.01b	0.03 ± 0.005b	0.07 ± 0.02a	0.05 ± 0.01ab	4.312	0.003
^{153}Eu	0.01 ± 0.002b	0.01 ± 0.001ab	0.01 ± 0.002ab	0.02 ± 0.003a	0.01 ± 0.001ab	3.473	0.011
^{157}Gd	0.04 ± 0.01b	0.04 ± 0.01b	0.03 ± 0.004b	0.07 ± 0.02a	0.05 ± 0.01ab	4.357	0.003
^{163}Dy	0.03 ± 0.01ab	0.02 ± 0.003b	0.02 ± 0.003b	0.04 ± 0.01a	0.03 ± 0.005ab	3.639	0.009
^{165}Ho	0.003 ± 0.001b	0.002 ± 0.0003b	0.002 ± 0.0002b	0.005 ± 0.001a	0.004 ± 0.001ab	4.7	0.002
^{166}Er	0.01 ± 0.003ab	0.01 ± 0.002b	0.01 ± 0.001b	0.02 ± 0.01a	0.02 ± 0.002ab	3.915	0.006
^{169}Tm	0.001 ± 0.0002ab	0.001 ± 0.0001b	0.001 ± 0.0001b	0.002 ± 0.0007a	0.001 ± 0.0002ab	4.011	0.005
^{172}Yb	0.01 ± 0.002ab	0.01 ± 0.001b	0.01 ± 0.001b	0.02 ± 0.003a	0.01 ± 0.002ab	4.364	0.003

Elements	HongHe (n=15)	KunMing (n=15)	PuEr (n=15)	QuJing (n=36)	WenShan (n=8)	F-value	P-value
^{200}Hg	0.003 ± 0.001a	0.003 ± 0.0002a	0.003 ± 0.0002a	0.004 ± 0.0004a	0.003 ± 0.0002a	1.44	0.228
^{202}Hg	0.003 ± 0.001a	0.003 ± 0.0002a	0.003 ± 0.0002a	0.004 ± 0.0004a	0.003 ± 0.0002a	2.047	0.095
^{205}Tl	0.02 ± 0.0032a	0.03 ± 0.01a	0.02 ± 0.001a	0.04 ± 0.02a	0.03 ± 0.01a	0.709	0.588
^{206}Pb	0.735 ± 0.16a	0.528 ± 0.06abc	0.277 ± 0.03c	0.612 ± 0.09ab	0.325 ± 0.06bc	3.111	0.020
^{207}Pb	0.711 ± 0.16a	0.526 ± 0.06abc	0.282 ± 0.03c	0.612 ± 0.09ab	0.305 ± 0.06bc	3.058	0.021
^{208}Pb	0.72 ± 0.16a	0.52 ± 0.09abc	0.28 ± 0.04c	0.61 ± 0.12ab	0.31 ± 0.08bc	3.054	0.022
^{232}Th	0.003 ± 0.001a	0.01 ± 0.003a	0.01 ± 0.002a	0.01 ± 0.01a	0.003 ± 0.001a	1.496	0.211
^{238}U	0.023 ± 0.007a	0.019 ± 0.0043ab	0.007 ± 0.0011b	0.023 ± 0.007a	0.016 ± 0.003ab	2.943	0.025

Table S4. Multi-element contents and comparison results using T'test for *P.notoginseng* of different cultivation models (mg/kg). T'test was used to determine significance, **** (P < 0.0001), *** (P < 0.001), ** (P < 0.01), * (P < 0.05), and ns (P > 0.05).

Elements	Field(n=65)	Forest(n=24)
^{23}Na	24.34 ± 0.95ns	24.91 ± 3.08
^{24}Mg	1563.74 ± 46.42****	2113.44 ± 112.7
^{27}Al	613.99 ± 42.6****	284.17 ± 23.21
^{39}K	24035.51 ± 352.59***	19709.39 ± 977.07
^{43}Ca	958.42 ± 22.56***	1196.26 ± 52.58
^{44}Ca	1320.97 ± 31.54***	1663.73 ± 75.39
^{51}V	1.59 ± 0.12****	0.56 ± 0.05
^{52}Cr	1.88 ± 0.21****	0.72 ± 0.07
^{53}Cr	1.8 ± 0.2****	0.68 ± 0.07
^{55}Mn	47.68 ± 2.61*	38.94 ± 3.25
^{56}Fe	410.92 ± 43.92****	104.32 ± 10.6
^{57}Fe	398.15 ± 42.44****	125.16 ± 8.9
^{59}Co	0.41 ± 0.03****	0.12 ± 0.01
^{60}Ni	1.95 ± 0.1ns	2.28 ± 0.21
^{63}Cu	3.79 ± 0.13ns	4.23 ± 0.25
^{65}Cu	3.73 ± 0.13ns	4.24 ± 0.25
^{66}Zn	13.39 ± 0.52***	20.42 ± 1.58
^{75}As	0.37 ± 0.04****	0.15 ± 0.02
^{78}Se	0.04 ± 0.004*	0.03 ± 0.003
^{88}Sr	6.68 ± 0.31ns	7.65 ± 0.91
^{89}Y	0.16 ± 0.02**	0.1 ± 0.01
^{97}Mo	0.08 ± 0.01ns	0.06 ± 0.01
^{98}Mo	0.08 ± 0.01ns	0.06 ± 0.01
^{107}Ag	0.005 ± 0.0003*	0.02 ± 0.01
^{111}Cd	0.38 ± 0.03****	0.16 ± 0.01
^{114}Cd	0.38 ± 0.03****	0.16 ± 0.01
^{118}Sn	0.13 ± 0.02ns	0.1 ± 0.02
^{123}Sb	0.11 ± 0.01***	0.07 ± 0.003
^{137}Ba	14.48 ± 0.47****	24.8 ± 1.83
^{139}La	0.26 ± 0.02****	0.15 ± 0.01
^{140}Ce	0.52 ± 0.04****	0.21 ± 0.03
^{141}Pr	0.05 ± 0.004****	0.02 ± 0.002
^{146}Nd	0.3 ± 0.03****	0.15 ± 0.01
^{147}Sm	0.06 ± 0.005****	0.03 ± 0.003
^{153}Eu	0.02 ± 0.001ns	0.01 ± 0.001
^{157}Gd	0.06 ± 0.005****	0.03 ± 0.002
^{163}Dy	0.04 ± 0.003****	0.02 ± 0.001

Elements	Field(n=65)	Forest(n=24)
^{165}Ho	0.004 ± 0.0004****	0.002 ± 0.0001
^{166}Er	0.019 ± 0.0019****	0.01 ± 0.0008
^{169}Tm	0.002 ± 0.0002****	0.001 ± 0.00005
^{172}Yb	0.015 ± 0.0014****	0.007 ± 0.0007
^{200}Hg	0.003 ± 0.0002ns	0.003 ± 0.0002
^{202}Hg	0.003 ± 0.0002ns	0.003 ± 0.0002
^{205}Tl	0.03 ± 0.01ns	0.03 ± 0.002
^{206}Pb	0.61 ± 0.05****	0.34 ± 0.03
^{207}Pb	0.61 ± 0.05****	0.34 ± 0.03
^{208}Pb	0.61 ± 0.05****	0.34 ± 0.03
^{232}Th	0.01 ± 0.002ns	0.01 ± 0.002
^{238}U	0.02 ± 0.002****	0.01 ± 0.001

Table S5. The allocation of sampling areas for the *P. notoginseng* in Yunnan province, China.

Bases	Time	Cultivation Model	Origins	Bases	Time	Cultivation Model	Origins
JianShui1-1	2019	Field	HongHe	ShiLin1-1	2020	Field	KunMing
JianShui1-2	2019	Field	HongHe	ShiLin1-2	2020	Field	KunMing
JianShui1-3	2019	Field	HongHe	ShiLin1-3	2020	Field	KunMing
JianShui2-1	2019	Field	HongHe	XuanWei1-1	2020	Field	QuJing
JianShui2-2	2019	Field	HongHe	XuanWei1-2	2020	Field	QuJing
JianShui2-3	2019	Field	HongHe	XuanWei1-3	2020	Field	QuJing
ShiZong1-1	2019	Field	QuJing	XuanWei2-1	2020	Field	QuJing
ShiZong1-2	2019	Field	QuJing	XuanWei2-2	2020	Field	QuJing
ShiZong1-3	2019	Field	QuJing	XuanWei2-3	2020	Field	QuJing
ZhanYi1-1	2019	Field	QuJing	ZhanYi1-1	2020	Field	QuJing
ZhanYi1-2	2019	Field	QuJing	ZhanYi1-2	2020	Field	QuJing
ZhanYi1-3	2019	Field	QuJing	ZhanYi1-3	2020	Field	QuJing
ZhanYi2-1	2019	Field	QuJing	LuLiang1-1	2020	Field	QuJing
ZhanYi2-2	2019	Field	QuJing	LuLiang1-2	2020	Field	QuJing
ZhanYi2-3	2019	Field	QuJing	LuLiang1-3	2020	Field	QuJing
XuanWei1-1	2019	Field	QuJing	MaLong1-1	2020	Field	QuJing
XuanWei1-2	2019	Field	QuJing	MaLong1-2	2020	Field	QuJing
XuanWei1-3	2019	Field	QuJing	MaLong1-3	2020	Field	QuJing
XuanWei2-1	2019	Field	QuJing	ShiZong1-1	2020	Field	QuJing
XuanWei2-2	2019	Field	QuJing	ShiZong1-2	2020	Field	QuJing
XuanWei2-3	2019	Field	QuJing	ShiZong1-3	2020	Field	QuJing
QiuBei1-1	2019	Field	WenShan	QiuBei1-1	2020	Field	WenShan

Bases	Time	Cultivation Model	Origins	Bases	Time	Cultivation Model	Origins
QiuBei1-2	2019	Field	WenShan	QiuBei1-2	2020	Field	WenShan
QiuBei1-3	2019	Field	WenShan	QiuBei1-3	2020	Field	WenShan
DaTangZi1-1	2019	Forest	PuEr	YanShan1-1	2020	Field	WenShan
DaTangZi1-2	2019	Forest	PuEr	YanShan1-2	2020	Field	WenShan
DaTangZi1-3	2019	Forest	PuEr	BaZi1-1	2020	Forest	PuEr
DaTangZi2-1	2019	Forest	PuEr	BaZi1-2	2020	Forest	PuEr
DaTangZi2-2	2019	Forest	PuEr	BaZi1-3	2020	Forest	PuEr
DaTangZi2-3	2019	Forest	PuEr	LaoMianZhai1-1	2020	Forest	PuEr
JianShui1-1	2020	Field	HongHe	LaoMianZhai1-2	2020	Forest	PuEr
JianShui1-2	2020	Field	HongHe	LaoMianZhai1-3	2020	Forest	PuEr
JianShui1-3	2020	Field	HongHe	LaoMianZhai2-1	2020	Forest	PuEr
MengZi1-1	2020	Field	HongHe	LaoMianZhai2-2	2020	Forest	PuEr
MengZi1-2	2020	Field	HongHe	LaoMianZhai2-3	2020	Forest	PuEr
MengZi1-3	2020	Field	HongHe	HuiZe1-1	2020	Forest	QuJing
ShiPing1-1	2020	Field	HongHe	HuiZe1-2	2020	Forest	QuJing
ShiPing1-2	2020	Field	HongHe	HuiZe1-3	2020	Forest	QuJing
ShiPing1-3	2020	Field	HongHe	XunDian1-1	2020	Forest	KunMing
XunDian3-1	2020	Field	KunMing	XunDian1-2	2020	Forest	KunMing
XunDian3-2	2020	Field	KunMing	XunDian1-3	2020	Forest	KunMing
XunDian3-3	2020	Field	KunMing	XunDian2-1	2020	Forest	KunMing
XunDian4-1	2020	Field	KunMing	XunDian2-2	2020	Forest	KunMing
XunDian4-2	2020	Field	KunMing	XunDian2-3	2020	Forest	KunMing
XunDian4-3	2020	Field	KunMing				

Table S6. Agilent 8800 ICP-MS/MS operating parameters.

Parameter	Operating condition
RF power	1500 W
Sampling depth	8.0 mm
Number of replicates	3
Stabilization time	10 s
Auxiliary gas flow rate	0.40 L/min
Makeup gas flow rate	0.20 L/min
Carrier gas flow rate	1.05 L/min
Spray chamber temperature	2.0 °C
Quadrupole bias V	-16 V
Octo pole bias V	-18 V
Internal standard	^{45}Sc, ^{72}Ge, ^{74}Ge, ^{115}In and ^{209}Bi
Isotopes	^{23}Na, ^{24}Mg, ^{39}K, ^{43}Ca, ^{44}Ca, ^{51}V, ^{52}Cr, ^{53}Cr, ^{55}Mn, ^{56}Fe, ^{57}Fe, ^{59}Co, ^{60}Ni, ^{63}Cu, ^{65}Cu, ^{66}Zn, ^{75}As, ^{78}Se, ^{88}Sr, ^{89}Y, ^{97}Mo, ^{98}Mo, ^{107}Ag, ^{111}Cd, ^{114}Cd, ^{118}Sn, ^{123}Sb, ^{137}Ba, ^{139}La, ^{140}Ce, ^{141}Pr, ^{146}Nd, ^{147}Sm, ^{153}Eu, ^{157}Gd, ^{163}Dy, ^{165}Ho, ^{166}Er, ^{169}Tm, ^{172}Yb, ^{200}Hg, ^{202}Hg, ^{205}Tl, ^{206}Pb, ^{207}Pb, ^{208}Pb, ^{232}Th and ^{238}U
